# Circumferential long-segment Barrett’s esophagus fully involved by adenocarcinoma resected by endoscopic submucosal dissection

**DOI:** 10.1055/a-2712-3711

**Published:** 2025-10-09

**Authors:** Ana Teresa Ferreira, João Pedro Paulo, Luísa Freitas Gonçalves, Sara Archer, Ricardo Marcos Pinto, Isabel Pedroto, Ricardo Küttner-Magalhães

**Affiliations:** 1 674892 Gastroenterology, Unidade Local de Saúde de Santo António EPE, Porto, Portugal

Barrett’s esophagus (BE) is a known precursor to esophageal adenocarcinoma (EAC). In recent years, endoscopic treatment of EAC has become increasingly important 1 2 . Although endoscopic mucosal resection and endoscopic submucosal dissection (ESD) are recognized treatments for BE-related neoplasia, ESD is generally reserved for selected, advanced lesions. 3 . We present a rare case of long-segment BE completely and circumferentially involved by EAC that was successfully treated with a single session of ESD.

A 66-year-old man who was a former smoker reported occasional heartburn over the preceding 6 years. An upper gastrointestinal endoscopy revealed a long circumferential BE segment (Prague classification C14M14), from 27 to 41 cm from the incisors, with loss of structure of the mucosal and glandular pattern shown under narrow-band imaging and with early loss of aceto-whitening along its entire length; biopsies were compatible with EAC.

An ESD was carried out with proximal and distal circumferential incisions. Underwater ESD was performed with two contralateral submucosal tunnels, leaving two longitudinal lateral bridges intact. The clip-with-line technique was then used to aid specimen traction, allowing lateral bridge dissection ( Video 1 ). Triamcinolone was injected to prevent stenosis. A full circumferential specimen of 150 mm in length was retrieved ( Fig. 1 ). Histological examination revealed an R0 resection of a 140-mm, m2 well-differentiated EAC, LV0.

Endoscopic submucosal dissection is successfully performed for long-segment Barrett’s esophagus fully involved by adenocarcinoma.Video 1

**Fig. 1:**
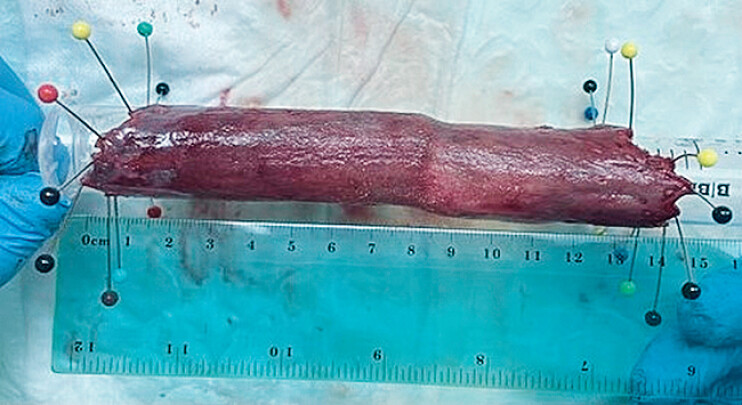
Photograph showing the final full circumferential resection specimen, measuring 150 mm in length.

The patient was discharged the day following the procedure on oral prednisolone. Despite systemic steroid therapy, esophageal stenosis developed, but was resolved through a program of esophageal dilations; 1 year after ESD, the patient is asymptomatic.

ESD for circumferential EAC within a long-segment BE, although complex, is a safe and effective procedure. We present a particularly demanding case in which the entire length of BE was occupied by EAC. In this case, while ESD was curative, it led to esophageal stenosis, reinforcing the value of enrolling patients undergoing circumferential esophageal ESD into regular prophylaxis or stenosis treatment programs.

Endoscopy_UCTN_Code_TTT_1AO_2AG_3AD

## Notes

Endoscopy E-Videos https://eref.thieme.de/e-videos
*E-Videos* is an open access online section of the journal *Endoscopy* , reporting on interesting cases and new techniques in gastroenterological endoscopy. All papers include a high-quality video and are published with a Creative Commons CC-BY license. Endoscopy E-Videos qualify for HINARI discounts and waivers and eligibility is automatically checked during the submission process. We grant 100% waivers to articles whose corresponding authors are based in Group A countries and 50% waivers to those who are based in Group B countries as classified by Research4Life (see: https://www.research4life.org/access/eligibility/ ). This section has its own submission website at https://mc.manuscriptcentral.com/e-videos .

## References

[1] Kaneko M Mitoro A Yoshida M Treatment of long-segment Barrett’s adenocarcinoma by complete circular endoscopic submucosal dissection: a case report BMC Gastroenterol 2018 18 16 10.1186/s12876-018-0743-9 29351773 PMC5775555

[2] Motomura D Bechara R Complete circumferential endoscopic submucosal dissection for early Barrettʼs neoplasia Gastrointest Endosc 2024 99 337 345 10.1016/j.gie.2023.09.008 37804873

[3] Subramaniam S Chedgy F Longcroft-Wheaton G Complex early Barrettʼs neoplasia at 3 Western centers: European Barrettʼs Endoscopic Submucosal Dissection Trial (E-BEST) Gastrointest Endosc 2017 86 608 618 10.1016/j.gie.2017.01.027 28159540

